# Preferential Involvement of BRCA1/BARD1, Not Tip60/Fe65, in DNA Double-Strand Break Repair in Presenilin-1 P117L Alzheimer Models

**DOI:** 10.1155/2022/3172861

**Published:** 2022-02-21

**Authors:** Marcella M. Authiat, Emmanuelle Gruz-Gibelli, Julien Colas, Estelle Bianchi, Marta Garcia-Arauzo, Pascale Marin, François R. Herrmann, Armand Savioz

**Affiliations:** ^1^Department of Psychiatry, Division of Geriatric Psychiatry, University Hospital Geneva, Geneva, Switzerland; ^2^VIVACY Laboratories, Archamps Technopole, Archamps, France; ^3^Department of Rehabilitation and Geriatrics, Division of Geriatrics, University Hospitals of Geneva and University of Geneva, Geneva, Switzerland; ^4^Geneva University Neurocenter, University of Geneva, Geneva, Switzerland

## Abstract

Recently, we showed that DNA double-strand breaks (DSBs) are increased by the A*β*_42_-amyloid peptide and decreased by all-trans retinoic acid (RA) in SH-SY5Y cells and C57BL/6J mice. The present work was aimed at investigating DSBs in cells and murine models of Alzheimer's disease carrying the *preseniline-1 (PS1) P117L* mutation. We observed that DSBs could hardly decrease following RA treatment in the mutated cells compared to the wild-type cells. The activation of the amyloidogenic pathway is proposed in the former case as A*β*_42_- and RA-dependent DSBs changes were reproduced by an *α*-secretase and a *γ*-secretase inhibitions, respectively. Unexpectedly, the *PS1 P117L* cells showed lower DSB levels than the controls. As the DSB repair proteins Tip60 and Fe65 were less expressed in the mutated cell nuclei, they do not appear to contribute to this difference. On the contrary, full-length BRCA1 and BARD1 proteins were significantly increased in the chromatin compartment of the mutated cells, suggesting that they decrease DSBs in the pathological situation. These Western blot data were corroborated by in situ proximity ligation assays: the numbers of BRCA1-BARD1, not of Fe65-Tip60 heterodimers, were increased only in the mutated cell nuclei. RA also enhanced the expression of BARD1 and of the 90 kDa BRCA1 isoform. The increased BRCA1 expression in the mutated cells can be related to the enhanced difficulty to inhibit this pathway by BRCA1 siRNA in these cells. Overall, our study suggests that at earlier stages of the disease, similarly to PS1 P117L cells, a compensatory mechanism exists that decreases DSB levels via an activation of the BRCA1/BARD1 pathway. This supports the importance of this pathway in neuroprotection against Alzheimer's disease.

## 1. Introduction

The *presenilin-1 (PS1) P117L* mutation is associated with one of the most severe forms of early-onset familial Alzheimer's disease (AD), with one patient deceased at the age of 28 y [1]. Disorientation was the first symptom observed, aside from memory loss [2]. In the SH-SY5Y cells, the *PS1 P117L* mutation did not alter the cellular morphology consistently [3], and when treated with all-trans retinoic acid (RA), these mutated cells differentiated normally [4] but potentiated the cell cycle arrest in the G1 phase [5]. In progenitor cells originating from embryos of *PS1 P117L* mice, we observed only faint impairments of cell differentiation [6]. In adult mice, neurogenesis was also altered in the hippocampus as a consequence of the *PS1 P117L* mutation [7] or due to the overexpression of the wild-type PS1 [8]. The *PS1 P117L* mice overproduced A*β*_42_, and not A*β*_40_ [7], but developed only few plaques and virtually no neurofibrillary tangles [9]. Furthermore, this mutation contributes to disinhibitory tendencies and a lower ability of mice to adapt to novel environments [9]. This ressembles the disoriented behavior of the *PS1 P117L* AD patients.

More recently, we have shown that A*β*_42_, but not A*β*_40_, induces DNA double-strand breaks (DSBs) which could be repaired or prevented by multiple RA-dependent pathways in the SH-SY5Y, in the astrocytic DI TNC_1_ cells, and in the C57BL/6J mice, indeed RA appears to repair DSBs and protect against A*β*_42_-induced DSBs [10, 11]. Such investigations have however not been carried out in *PS1 P117L* cell or mice models. We were interested to analyze, in the present study, DSBs in *PS1 P117L* cells and mice compared to the SH-SY5Y cells and B6D2 wild-type mice, knowing especially the particularly strong deleterious effect of this mutation. They were firstly investigated in relation to RA and to the amyloid cascade by *α*-, *β*-, and *γ*-secretase inhibitors to increase or decrease DSB production in the mutated relatively to the wild-type models. The observation of DSB levels that were lower in the mutated cells than in the wild-type cells—contrarily to what was expected due to higher endogenous A*β* production in the mutated models—led us to investigate two DSB repair pathways. One involves the Fe65 and Tip60 DSB repair factors and the other the breast cancer antigen 1 protein (BRCA1) and BRCA1-associated ring domain protein 1 (BARD1). Such pathways might be overactivated in the mutated models due to compensatory mechanisms.

For the Tip60/Fe65 pathway, various models have been proposed [12, 13]. A consensus has been reached, suggesting that the internal C-terminal domain of the amyloid precursor protein or AICD, in interaction with Fe65, is translocated to the cell nucleus [13]. In this cell compartment, it would form a transcriptively active complex [14] with the histone acetyl transferase Tip60 [15, 16]. This complex would be recruited at DSB sites [17]. Increased Tip60 expression would promote DNA repair via, for instance, acetylation of H3K14 [18], a factor that is increased in the occipital cortex of postmortem AD [19]. In addition, phosphorylation of Tip60 would increase histone acetyl transferase activity [20].

In regard to the BRCA1/BARD1 pathway, a decrease of A*β*_42_-induced DSBs is related to an enhanced expression of BRCA1 in AD [21]. The observation of an increased BRCA1 immunoreactivity in AD histopathological lesions was also corroborated [22]. Indeed, colocalization of BRCA1 with neurofibrillary tangles (NFTs) is already known [23]. BRCA1 is upregulated to induce DSB repair and is mislocated in advanced stages of AD to cytoplasmic NFTs, leading to an accumulation of DNA fragmentation [24]. BRCA1 is also known to enhance DNA damage response associated with neurons in the cell cycle entry and ultimately in apoptosis [25, 26]. BARD1 by forming a heterodimer with BRCA1, via its N-terminal RING domain, favors its migration into the nucleus. By blocking the nuclear export signal of BRCA1, the heterodimers are retained in the nuclear compartment where they contribute to the DNA repair and the transcription activation [27–29]. In the cytoplasm, BRCA1 and BARD1 are independently implicated in apoptosis [30]. The C-terminal BRCT domains of BRCA1 were shown to interact with LMO4 and to repress the BRCA1-mediated transcriptional activation, thus possibly diminishing DNA repair and cell cycle control [31]. Phosphorylation of BRCA1, at the serine 1524, recruits repair proteins of DSBs [32].

The subcellular localization of proteins of both pathways, i.e., Fe65, Tip60, and BRCA1, can be altered by leptomycin B, an inhibitor of nuclear export [27, 33], and the phosphorylation of Tip60 can be prevented by roscovitin, a CdK5 inhibitor [33]. Both compounds were used to decrease DSB repair.

Overall, the aim of this study was to investigate the difference in DSB levels between AD cellular and mice models and their controls as well as to determine the DSB repair pathways involved. For the animal studies, we separated the neocortex in the superficial and deep neocortical layers as the former appear to be preferentially vulnerable especially to the A*β* deposits [34, 35] and less so to the NFT deposits [36, 37], allowing possibly to related cortical vulnerability to one of these pathways.

We observed that the *PS1 P117L* mutation results, contrarily to the wild-type case, in increased DSBs following various treatments (e.g., leptomycin B and *α*-secretase inhibitor) and in almost no decreased DSBs when treated with RA. The lower DSB levels observed in the mutated cells compared to the wild-type cells appear, as shown by Western blots and by in situ proximity ligation assays, not to be due to the Fe65 and Tip60 proteins, which were preferentially expressed in the wild-type cells, but to be due to the enhanced expression of the BRCA1 and BARD1 proteins in the cell nuclei of the PS1 P117L cells, resulting in an increase of BRCA1-BARD1 heterodimers and of resistance to BRCA1 siRNA inhibition in the mutated compared to the control cells.

## 2. Materials and Methods

### 2.1. Culture of the SH-SY5Y Cells

Human SH-SY5Y cells (European Collection of Animal Cell Culture, UK) were routinely grown at 37°C in RPMI-1640 (Gibco Life Technologies) with 10% FCS (Bioconcept, Switzerland), 2 mM L-glutamine (Gibco Life Technologies), 100 IU/ml penicillin G, and 100 *μ*g/ml streptomycin (Invitrogen) in an incubator containing 5% CO_2_, 95% humidified air. Cells were passaged every 5 to 6 days. For this purpose, the confluent cells were released in DPBS (150 mM NaCl, 3 mM KCl, 1.5 mM KH_2_PO_4_, 7.9 mM Na_2_HPO_4_·2H_2_O, and 0.1 EDTA; pH 7.4), centrifuged at 1000 rpm during 5 min, and resuspended in RPMI-1640 and 10% FCS, at the desired dilution.

The cells were photographed with an inverted phase contrast microscope (Z*eissTELAVAL* 31) using a photo camera (Leica DFC 490) and the FireCam analyst software (Leica).

### 2.2. Production of the SH-SY5Y Clones with Wild-Type *PS1* or Mutated *PS1 P117L* Transgenes

For the transfection of the SH-SY5Y cells, various pcDNA3 plasmid (Invitrogen) constructs with the wild-type (Wt) presenilin-1 (PS1) or the mutated PS1 P117L genes were used. The plasmid pcDNA3::PS1 was from Pigino et al. [38] and the pcDNA3::PS1 P117L from Dowjat et al. [39]. The presenilin-1 inserts were verified by sequencing with primers PS1-F (5′-489-CATGTGATCATGCTCTTTGT-508-3′ XM_007441) and PS1-R1 (5′-1552-AGAGCTGGCAATGCTTTCTT-1533-3′ XM_007441, 20 ng/*μ*l) (DNA Sequencing Facility, CMU, Uni., Geneva). The extracted plasmids (Plasmid Mini Kit, Qiagen) were linearized with PvuI (Invitrogen) and purified with the “High Pure PCR Product Purification Kit” (Roche). 70% confluent SH-SY5Y cells were transfected using the DOTAP liposomal transfection reagent, according to the manufacturer's protocol (Roche). They were resuspended after 48 h in a selective medium containing Geneticin at 400 *μ*g/ml for 10 days. The concentration was then reduced to 200 *μ*g/ml, and about 10 clones were isolated for each construct. The genomic insertion of the constructs was verified by amplification (Ready-to-go RT-PCR beads, Amersham Biosciences) of TRIzol (Invitrogen)-extracted RNA. Random hexamers (Amersham Biosciences) were used for the reverse transcription. For the PCR amplifications of the PS1 (760 base pair (bp)) or PS1 P117L (960 bp) inserts, T7 primer (5′-TAATACGACTCACTATAGG-5′) and PS1-R2 primer (5′-797-CCCCAAGTAAATGAATGAA-779-3′ XM_007441) were used. T7 and SP6 (5′-GATTTAGGTGACACTATAG-3′) primers were used for the amplification of the pcDNA3 plasmid alone (160 bp). Fragment visualization was carried out on agarose MP gels (Roche). A subset of positively transfected cells was verified by Western blots for enhanced PS1 expression in comparison to Wt cells and to clone pc11, with the transfection of the pcDNA3 vector.

### 2.3. PS1 P117L and B6D2 Mice, Tissue Samples, and Dissections

PS1 P117L mice and their Wt B6D2 littermates [9] were sacrificed at 1.5-6 months (young adults) or at 17-18 months (aged adults) using CO_2_ in accordance with the Swiss veterinary regulations. Mice were either female (comet assay) or male (Western blots).

Brains were quickly withdrawn. After extraction of the hippocampus and the frontal cortex, the most caudal part of the neocortex was removed, and the superficial (I–III) and deep (V-VI) layers of the medial neocortex were immediately separated under a binocular microscope at the level of layer IV, weighted and minced. The superficial neocortical layers are known to be preferentially vulnerable to A*β* deposits as explained in the Introduction.

### 2.4. Tissue and Cell Treatments with Chemicals and siRNAs

For the cell treatments, two days before the experiment, the RPMI-1640 medium with 10% FCS was replaced by RPMI-1640 with 1% FCS, and a cell number appropriate to each method was used. For the cortical tissue treatment, 1 mg was utilized in neurobasal medium (Gibco Life Technologies), without NB27, in the presence of penicillin G, streptomycin, and L-glutamine. The cultured cells and tissues were treated or not, at 37°C for 30 min or 1 h in a CO_2_ incubator, with 20 *μ*M monomeric A*β*_42_ peptides (Enzo Life Sciences), 100 nM leptomycin B (Sigma-Aldrich), 5 *μ*M all-trans retinoic acid (RA, Sigma-Aldrich), 20 *μ*M roscovitin (Sigma-Aldrich), 10 *μ*M *α*-secretase inhibitor (Labforce), 50 nM *β*-secretase inhibitor, or 13.5 *μ*M *γ*-secretase inhibitor (Sigma-Aldrich). All treatments lasted for short times to avoid cell death. Leptomycin B, by inhibiting the CRM1 nuclear membrane protein, blocks nuclear export [40] and inactivates Tip60 [33]. Leptomycin B also suppresses BRCA1 recruitment to the DNA to repair it and its return to the cytoplasm [27]. Roscovitin, a CdK5 inhibitor, blocks DSB repair by preventing the phosphorylation of Tip60 [33].

For the siRNA treatment, 10 to 20 *μ*l X-tremeGENE siRNA transfection reagent (Roche, Mannheim) diluted in 50 *μ*l RPMI was added to 120 pmol or 240 pmol BRCA1 siRNA (Cell Signaling) or ADAM10 siRNA (Santa Cruz), or not, in a final volume of 300 *μ*l and then was added to the cells for 2 h in a CO_2_ incubator. 2.5 *μ*l siRNA with scrambled sequences in 100 *μ*l RPMI was used as control according to the manufacturer (Santa Cruz).

### 2.5. Neutral Single-Cell Gel Electrophoresis

DSBs were measured in the SH-SY5Y cells and cells originating from the homogenized murine cortex, using the Trevigen CometAssay^TM^ kit (AMS Biotechnology, UK) with the following modifications. After treatment, the SH-SY5Y cells were resuspended in Ca^2+^- and Mg^2+^-free PBS at a concentration of 1.0 to 1.5 × 10^5^ cells/ml. An identical number of cortical cells was resuspended in the same solution with the addition of ice-cold 20 mM EDTA. An aliquot of 50 *μ*l SH-SY5Y or cortical cells was added to 500 *μ*l of 1% molten low-melting agarose (SeaPlaque, FMC BioProduct, USA) kept at 42°C. 50 *μ*l was immediately spread on a comet slide (AMS Biotechnology), which was incubated at 4°C in the dark for 10 min to accelerate agarose gelling. The slide was then transferred to a prechilled lysis solution (AMS Biotechnology) for 60 min at 4°C. Subsequently, the slide was incubated in neutral electrophoresis buffer (500 mM Tris base, 1.5 M sodium acetate, pH 9.0) for 30 min at 4°C. The DNA fragments were separated by electrophoresis at 26 V for 45 min. Then, the slide was immersed in 70% ethanol at room temperature (RT) for 30 min and was air dried. The DNA was stained 10 min at RT with 100 *μ*l SYBR Green I dye (Gibco Life Technologies) diluted 1 : 1000 in water and then rinsed with distilled water. Immediately afterwards, at least 30 comets per treatment were photographed using an Olympus digital camera attached to an epifluorescent Zeiss Axioplan microscope (Axio vision rel 4.6) and analyzed for comet tail length (see 2.8).

### 2.6. Western Blots and Subcellular Fractionation

The SH-SY5Y cells or clones were centrifuged at 1000 rpm for 5 min and resuspended in a standard buffer containing antiproteases and okadaic acid and then sonicated 2 × 10 sec or fractionated with the Subcellular Protein Fractionation kit (Life Technologies). 1.0 to 3.0 × 10^6^ cells were used to extract cytoplasmic, nuclear, and chromatin proteins. Protein concentration was determined by the BCA protein assay (Pierce, Thermo Scientific), and proteins were denatured in standard Laemmli loading buffer. 5-10 *μ*g of the fractionated proteins and 20 *μ*g of the nonfractionated proteins were denatured (100°C, 5 min), diluted in Laemmli loading buffer, separated by 7.5% SDS-polyacrylamide gel electrophoresis (Ready gel 7.5%, Mini-Protean Tetra System, Bio-Rad, 60 V, 1 h and then 90 V, 1 h), and electroblotted (overnight, 35 V; Mini Trans-Blot electrophoretic transfer cell, Bio-Rad) to a nitrocellulose membrane (Protran BA85, Schleicher and Schuell), according to a standard Western blot method [6]. 6.5 *μ*l Seeblue Plus 2 (Invitrogen) was loaded as a molecular weight prestained standard. The membrane was incubated overnight at 4°C in PBS with 0.2% Tween 20, 0.5% BSA, and 5% milk with the following primary rabbit polyclonal antibodies: anti-BARD1 (A300-263A, BL, Bethyl Lubioscience), anti-histone H3 (acetyl K14 [EP964Y], ab52946, Abcam; 1 : 2000), anti-presenilin-1 (against the N-terminus, H-70, sc-7860, Santa Cruz Biotechnology), anti-presenilin-1 (against the C-terminus, AB5308, Chemicon and P7854, Sigma), anti-synaptophysin (SAB4502906, Sigma), anti-Tip60 (SAB45000118, Sigma), and anti-phospho-Tip60 (pSer86, SAB4504108, Sigma). The following primary mouse monoclonal antibodies were also used: AD2 (Bio-Rad; 1 : 500), AT8 (MN1020, Thermo Fisher Scientific), anti-BRCA1 OP92 (MS110, Sigma-Aldrich; 1 : 60), Tau-1 (MAB3420, Millipore; 1 : 200), and anti-Fe65 (against the N-terminus, sc398389, Santa Cruz Biotechnology). The goat anti-Fe65 (against the C-terminus, ab51980, Abcam) was also used. The anti-LMO4 was from Santa Cruz (goat, C15, sc-11122) or from Abcam (rabbit, ab229226). All primary antibodies were diluted 1 : 1000, except when specified otherways. Specific bands were detected by chemiluminescence (Amersham ECL™ Western Blotting Detection Reagent, GE Healthcare) on Hyperfilm ECL (Amersham Biosciences, GE Healthcare) with horseradish peroxidase-labeled anti-rabbit, anti-mouse, or anti-goat antibodies (DakoCytomation; diluted 1 : 3000 in PBS with 0.2% Tween 20, 0.5% BSA, and 5% milk). Loading of equal amounts of proteins was verified by using a mouse monoclonal anti-GAPDH antibody (Millipore; 1 : 10000) and by DB71 membrane staining (Aldrich Chemical Company, USA) for the fractionated cells. The ratios of the signals for a defined protein, measured by densitometry (U:Genius 3 with GeneTools from Syngene), divided by GAPDH or DB71 signals, were used for statistical analysis.

### 2.7. Duolink™ In Situ Proximity Ligation Assay

For the *in situ* proximity ligation assay (PLA, Duolink® In Situ Fluorescence, Sigma-Aldrich), 40,000 cells were seeded in each well, from a 12 well plate, containing each time a 15 mm circular, alcohol sterilized coverslip, and proliferation medium. Once the cells reached 80% confluence, they were treated or not, as described previously, washed in PBS 1X, and fixed with paraformaldehyde 4% for 20 min. Then, the cells were covered with one drop of blocking solution (Sigma-Aldrich) per 1 cm^2^, and the samples were incubated in a 37°C preheated humidity chamber for 30 min. The primary antibodies used as pairs were diluted in antibody diluent (Sigma-Aldrich) at 1 : 100. The pairs were the following ones: the rabbit anti-BARD1 BL with the mouse anti-BRCA1 OP92, the goat anti-LMO4 C15 with the mouse anti-BRCA1 OP92, the goat anti-Fe65 with the rabbit anti-Tip60, and the goat anti-Fe65 with the rabbit anti-phospho-Tip60. The manufacturers of these antibodies are listed in Section 2.6.

The blocking solution was tapped off, and 200 *μ*l of the mix of primary antibodies was added to each coverslip that was incubated 1 h30 in a humidity chamber at RT. For the negative control, no primary antibodies were added. Subsequently, the coverslips were washed twice in 1X Wash buffer A (DUO82047 Sigma-Aldrich) for 5 min, and 80 *μ*l of both PLA probes, PLA PLUS and PLA MINUS, diluted 1 : 5 in antibody diluent, was added onto each coverslip after the removal of the primary antibodies. The samples were incubated in a 37°C preheated humidity chamber. After 1 h, the PLA probes were tapped off; the cells were washed twice, 5 min in 1X Wash buffer A under gentle agitation and ligases, diluted 1 : 40 in ligation solution (diluted 1 : 5 in water), added to each well, and incubated in a 37°C preheated humidity chamber for 30 min. After removal of the ligation solution, the cells were washed twice for 2 min in 1X Wash buffer A, under gentle agitation. Then, polymerases, diluted 1 : 80 (10 units/*μ*l) in the amplification solution (diluted 1 : 5 in water), were added to each well, and the samples were incubated in a 37°C preheated humidity chamber for 1 h30. Next, the samples were washed in the dark twice for 5 min with 1X Wash buffer A, once for 30 sec with 0.01X Wash buffer B (DUO82048), and dried at RT in the dark. Then, the cells were colored with DAPI, diluted 1 : 5000 in PBS 1X. The coverslips were mounted on degreased alcohol slides with FluorSave reagent, and after 15 min, they were analyzed with an epifluorescent Zeiss Axioplan microscope. Pictures were taken using the AxioVision software. They were magnified 40x using a green filter (450-500 nm) and were imported into MetaMorph software for analyses. Alternatively, a Leica microscope (Leica DFC 7000 T) was used with its software (LAS X Life Science), and the jpg pictures were analyzed with ImageJ.

### 2.8. Statistical Analyses

For the comet assay, values of mean comet tail length were compared for each condition by a one-way analysis of variance (ANOVA), to establish the effects of various treatments. When overall statistically significant differences in treatments effect were obtained by ANOVA, comparisons of means among subgroups were made with Bonferroni multiple comparisons test.

In parallel, the nonparametric Kruskal-Wallis test was used to compare the shape of comet tail distribution, along with Bonferroni corrections to compare subgroups. The Kruskal-Wallis analysis is particularly suitable when the number of measures is small (less than 15 per group) or when the distribution is not Gaussian (asymmetric box plot).

For the Western blots and the in situ proximity ligation assay with the cells, an ANOVA test per group was followed by Tukey's and Sidak's multiple comparison tests, respectively, to compare the treatments between groups (SH-SY5Y and PS1-P117L cells). With the mice, protein expression was compared using two-way repeated measures ANOVA, with age (young versus old mice) and treatment effect (without RA, 1 h RA, and 2 h RA) along with their interaction. Treatment was the within-subject factor. The same analysis was performed separately for measures in the external or internal cortex layers, for each of the proteins and for each control used (DB71 or GAPDH). The significance values were determined using the Greenhouse–Geisser method.

## 3. Results

### 3.1. Characterisation of the SH-SY5Y Clones with the *PS1* or *PS1 P117L* Genes

**Figure 1:**
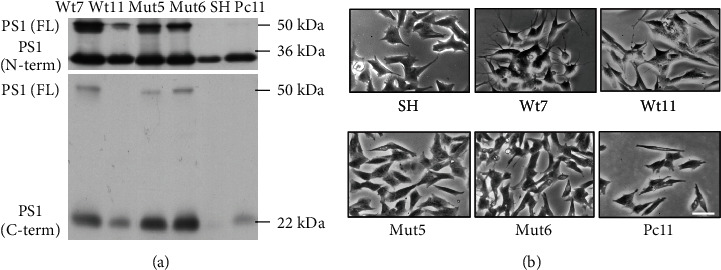
SHSY-5Y cells transfected with plasmid constructs with the presenilin-1 (PS1) gene with the PS1 P117L mutation or not. Some clones with the wild-type (Wt) or the mutated (Mut) PS1 gene were selected and characterized by Western blots (a). The full-length (FL) PS1 protein was overexpressed in the clones compared to the control SH-SY5Y cells (SH) and the clone transfected with the pcDNA3 vector alone (Pc11). FL PS1 is cleaved in two as observed with antibodies against the PS1 N-terminus (N-term) or the C-terminus (C-term). The overexpression was weaker in the Wt11 clone. (b) The Wt and Mut clones could not be distinguished according to their morphological structures, as observed by phase contrast microscopy. Scale bar: 200 *μ*M.

Ten clones per transfection were verified for plasmid insertion by RT-PCR (results not shown). A subgroup was checked by Western blots. Three clones with the *wild-type PS1* gene, Wt4, Wt7, and Wt11, and two with the mutated *PS1 P117L* gene, Mut5 and Mut6, were used in this study. They all showed an enhanced expression of the full-length (FL) PS1 protein (50 kDa), in comparison to the SH-SY5Y cells and to Pc11 clone, with the transfected vector pcDNA3 (Figure 1(a)). The other signals correspond to the N-terminus (expected 28 kDa) or the C-terminus (expected 18 kDa) of the PS1 protein, respectively. They result from the endoproteolytic processing of the FL PS1, independently from the mutation, as already shown by others [41, 42]. The clone Wt4 (not shown) had a similar pattern of expression as Wt7. Furthermore, the genotype was not related to morphological cell features, such as neuritic outgrowth or cell aggregation (Figure 1(b)), corroborating the data in SH-SY5Y [3] and in N2a cells [39]. Mut clones divided less and died about 2.4 times more than the SH-SY5Y cells, as assessed by trypan blue cell staining. Despite these variations, the PS1 clones all behaved differently from the PS1 P117L clones in the experiments described below. This means that the effect of the PS1 P117L mutation predominates on the differences in PS1 expression, or in cell phenotype and cell division.

### 3.2. DNA Double-Strand Breaks in the PS1 and PS1 P117L Clones

**Figure 2:**
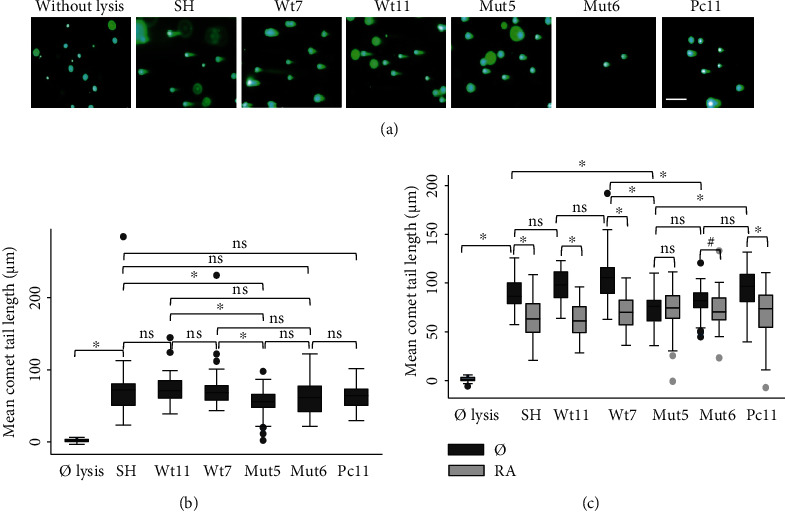
Comet tail lengths or DNA double-strand breaks (a) in the SH-SY5Y cells (SH), in the clones with the PS1 human gene (Wt7 and Wt11), with the P117L PS1-mutated gene (Mut5 and Mut6), and with the transfected vector pcDNA3 alone (Pc11) in comparison to cells without lysis. Scale bar: 200 *μ*M. (b) Box plots of mean comet tail lengths (in *μ*M) of SH cells and Wt and Mut clones (number of cells measured: 31 < *n* < 56). Comet tail lengths were significantly shorter in the Mut clones compared to the Wt clones. (c) 5 *μ*M RA treatment for 30 min reduced tail length particularly in the Wt cells and less so in the Mut clones (number of cells measured: 30 < *n* < 58) including the Mut6 clone (^#^*p* = 0.058/mean and *p* = 0.03/variance Kruskal). ANOVA with Bonferroni test: ^∗^*p* < 0.05; ns: nonsignificant.

Measurements of the comet tail lengths of the Wt clones compared to the Mut clones revealed that they were never longer in the latter (Figures 2(a) and 2(b)). On the contrary, they showed a significant decrease or a decreasing trend in the Mut clones. This constant lack of increase in DSBs was surprising as the increase of endogenous A*β*_42_ expression, due to the *PS1 P117L* mutation, was expected to increase the DSB levels. A 30 min treatment with all-trans retinoic acid (RA) decreased DSBs more efficiently in the Wt than the Mut clones, with Mut5 showing no decrease at all (Figure 2(c)). The Wt clones treated with RA resulted in similar DSB levels as the untreated Mut clones.

### 3.3. Inhibition of the *β*-Amyloid Cascade in the PS1 and PS1 P117L Clones

**Figure 3:**
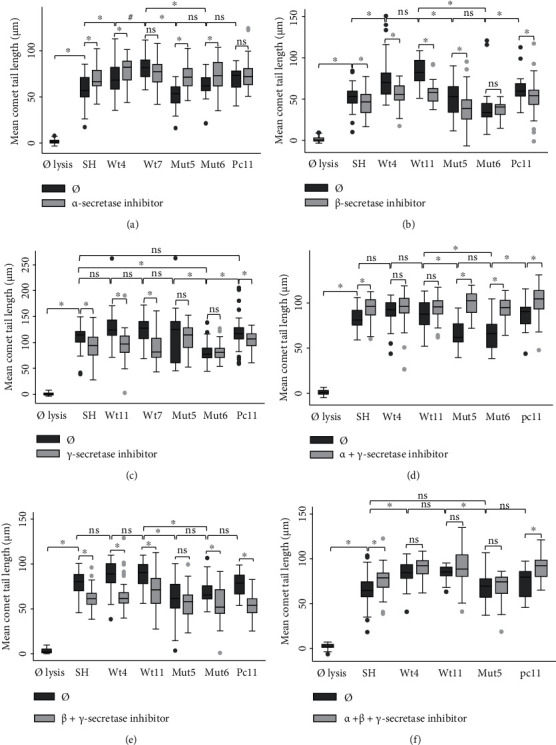
Comet tail lengths (in *μ*M) or DNA double-strand breaks (DSBs) and the *β*-amyloid cascade. The cleavage of APP was altered by (a) an *α*-secretase inhibitor (10 *μ*M, 1 h), by (b) a *β*-secretase inhibitor (50 nM, 1 h), by (c) a *γ*-secretase inhibitor (13.5 *μ*M, 1 h), or by a combination of inhibitors ((d) *α*- + *γ*-secretase inhibitors, 1 h; (e) *β*- + *γ*-secretase inhibitors, 1 h; and (f) *α*- + *β*- + *γ*-secretase inhibitors, 1 h). The *α*-secretase inhibition generally resulted in increased DSBs, whereas the *β*-secretase and the *γ*-secretase inhibitions decreased DSBs mainly in the nonmutated cells. The *α*- + *γ*-secretase inhibitors resulted in increased DSBs, except in the Wt clones. The *β*- + *γ*-secretase inhibitors generally resulted in decreased DSBs, whereas the *α*- + *β*- + *γ*-secretase inhibitors resulted in no DSB changes in the Wt and the Mut clones. Number of cells measured: 19 < *n* < 47. ANOVA with Bonferroni test: ^∗^*p* < 0.05; ^#^*p* < 0.001; ns: nonsignificant (Kruskal-Wallis).

We then decided to investigate if, by inhibiting the *α*-, *β*-, and/or *γ*-secretases, the three main enzymes of the *β*-amyloid cascade, the DSB levels vary between Wt and Mut clones (Figure 3). The inhibition of *α*-secretase (10 *μ*M, 1 h; Figure 3(a)) should result in increased endogenous A*β* and thus in increased DSBs. As expected, a significant increase of DSBs was observed in all cases, except for Wt7 and Pc11. The increase was more pronounced in the Mut clones than in the Wt clones (16 versus 10 *μ*m). On the contrary, the inhibition of *β*-secretase (50 nM, 1 h; Figure 3(b)) should decrease A*β* production and thus DSBs. This was the case except for the Mut6 clone. The decrease in comet tail length was more pronounced in the Wt clones than in the Mut5 clone (22 versus 6 *μ*m). For the *γ*-secretase inhibitor or presenilin-1 inhibitor (13.5 *μ*M, 30 min; Figure 3(c)), a significant decrease of DSBs was also expected. The comet tail lengths were about 36 *μ*m shorter with the Wt clones, but not with the Mut clones, where a background DSB level appears to have been reached. Alternatively, the inhibition of the mutated *γ*-secretase in the Mut clones might be impaired due to conformational changes. The combined inhibition of *α*- + *γ*-secretase (1 h, Figure 3(d)) showed a similar pattern to the *α*-secretase inhibition with a more pronounced increase in the Mut clones than in the Wt clones (33 *μ*m versus 6 *μ*m). This suggests that the *γ*-secretase inhibition might be less efficient than the *α*-secretase inhibition. The combined inhibition of *β*- + *γ*-secretase (1 h, Figure 3(e)) showed a consistent decrease of DSBs with a nonsignificant decrease in the Mut5 clone only. The decrease was more pronounced in the Wt than in the Mut clones (19 versus 10 *μ*m), similarly to the *β*-secretase or *γ*-secretase inhibitions. Inhibition of *α*- + *β*- + *γ*-secretases resulted in no DSB changes in Wt and Mut cells, except in the SHSY-5Y and Pc11 cells, due to the inhibition of enzymes with opposite effects.

Overall, these data demonstrate that the *α*- and the *β*-/*γ*-secretase pathways contribute in their own way to DSBs production, via the activation or not of the *β*-amyloid cascade.

### 3.4. Impairing DSB Repair by Blocking Nuclear Export in the PS1 and P117L PS1 Clones

**Figure 4:**
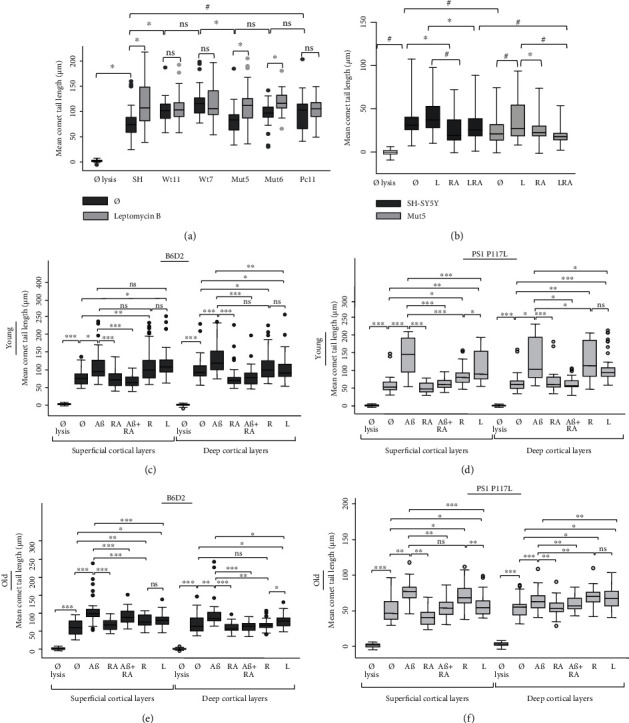
In (a), the SH-SY5Y (SH) cells, the wild-type (Wt) clones, and the mutated (Mut) clones were treated for 30 min with 100 nM leptomycin B (L) or not. The treatment increased the comet tail length (in *μ*M), particularly in the Mut clones (and the SH cells) compared to the Wt clones or the control Pc11 clone (number of cells measured: 33 < *n* < 46). In (b), compared to the untreated cells, leptomycin B (100 nM, 30 min) was shown to increase the comet tail length in the Mut5 cells. The all-trans retinoic acid (RA) was shown to decrease DSBs in the SH cells. RA was shown to significantly decrease DSBs in the presence of leptomycin B (LRA) compared to the leptomycin B treatment, especially in the Mut5 cells (number of cells measured: 90 < *n* < 97). In (a) and (b), ANOVA with Bonferroni multiple comparisons test: ^∗^*p* < 0.05; ^#^*p* < 0.001; ns: nonsignificant (Kruskal-Wallis). (c–f) DSBs in the superficial and deep cortical layers of 5-6-month (c and d) or 18-month (e and f) B6D2 (c and e) and PS1 P117L (d and f) female mice (*n* = 3 per group c, d, e, or f). The cortical layers were dissected and treated or not with 20 *μ*M A*β*, 5 *μ*M all-trans retinoic acid (RA), A*β*+RA, 20 *μ*M roscovitin (R), and 100 nM leptomycin B (L) for 30 min. We observed that the increased numbers of DSBs by A*β* was usually significantly decreased by the presence of RA. An effect of the strains could also be shown. Comet tails were longer in the PS1 P117L mice treated with roscovitin or leptomycin B compared to the B6D2 mice, for the deep cortical layers and the young mice only. ns: nonsignificant; ^∗^*p* < 0.05 for 1 mouse; ^∗∗^*p* < 0.05 for 2 mice; ^∗∗∗^*p* < 0.05 for 3 mice.

To study the difference in DSB levels between the Wt and the Mut clones, we disrupted the DSB repair pathways, such as the one involving Tip60/Fe65 or BRCA1, by treating the cells with 100 nM leptomycin B, for 30 min. The Wt and the Pc11 clones showed no increase of DSBs, compared to the Mut clones (Figure 4(a)). The comet tail lengths grew 23 *μ*m in the Mut clones and only 3 *μ*m in the Wt clones. Thus, leptomycin B appears to prevent DSB repair, particularly in the Mut clones. The experiment of Figure 4(b) showed that leptomycin B was more efficient to increase DSBs in the Mut5 cells than the Wt cells. Wt cells either significantly increased DSBs (Figure 4(a)) or did not (Figure 4(b)). Furthermore, RA could significantly reduce DSB increase when used with leptomycin B. The decrease was more significant for the Mut5 cells than for the SH-SY5Y cells. The treatment with roscovitin (1 h, 20 *μ*M) increased DSBs in the Mut clones but not in the SH-SY5Y and the Wt cells (results not shown), similarly to the results obtained with leptomycin B. These data suggest that, in the condition without treatment, at least one DSB repair pathway must be activated in the Mut clones. This pathway could lower the levels of DSBs preferentially in the Mut clones and would be altered by treatments with leptomycin B or with roscovitin.

### 3.5. DSBs in the Cortical Layers of Young and Old B6D2 and PS1 P117L Mice

To verify whether the effects of leptomycin B and roscovitin could be reproduced in mice, the cortical tissues of 6 B6D2 mice, 3 young and 3 old, and of 6 PS1 P117L mice, 3 young and 3 old, were separated immediately after being sacrificed in superficial and deep cortical layers and treated, or not, with A*β*_42_, RA, A*β*_42_ + RA, roscovitin, and leptomycin B (Figures 4(c)–4(f)). The young mice were 5-6 months of age and the old mice 18 months. We expected a decrease of the A*β*-induced DSBs in the presence of RA and an increase with A*β*_42_, roscovitin, or leptomycin B. Indeed, RA treatment resulted mostly in a significant decrease of DSBs and A*β*_42_ in longer comet tail lengths. The treatments with roscovitin or leptomycin B generated statistically significant increases in DSBs in the deep cortical tissue of 2/3 or 100% of the young PS1 P117L mice, respectively (Figure 4(d)). However, it reached significance in only 1/3 of the young B6D2 mice for the same tissue (Figure 4(c)). This effect was not observed in the superficial cortical layers of young mice and in all layers of old mice. It corresponds to the important increase of DSBs in the Mut clones, compared to the SH-SY5Y cells following the same treatments (Figure 4(b)). Furthermore, similarly to the untreated Mut and Wt clones, the DSB levels were significantly lower in the untreated PS1 P117L mice compared to the untreated B6D2 mice, however only in the superficial layers of the young mice. This difference should be corroborated with a larger number of mice. Finally, it is worth noting that the comet tail lengths were especially short in the old PS1 P117L mice.

### 3.6. Tip60, Fe65, and H3K14 Protein Expressions in Subcellular Compartments of the SH-SY5Y and PS1 P117L Cells

**Figure 5:**
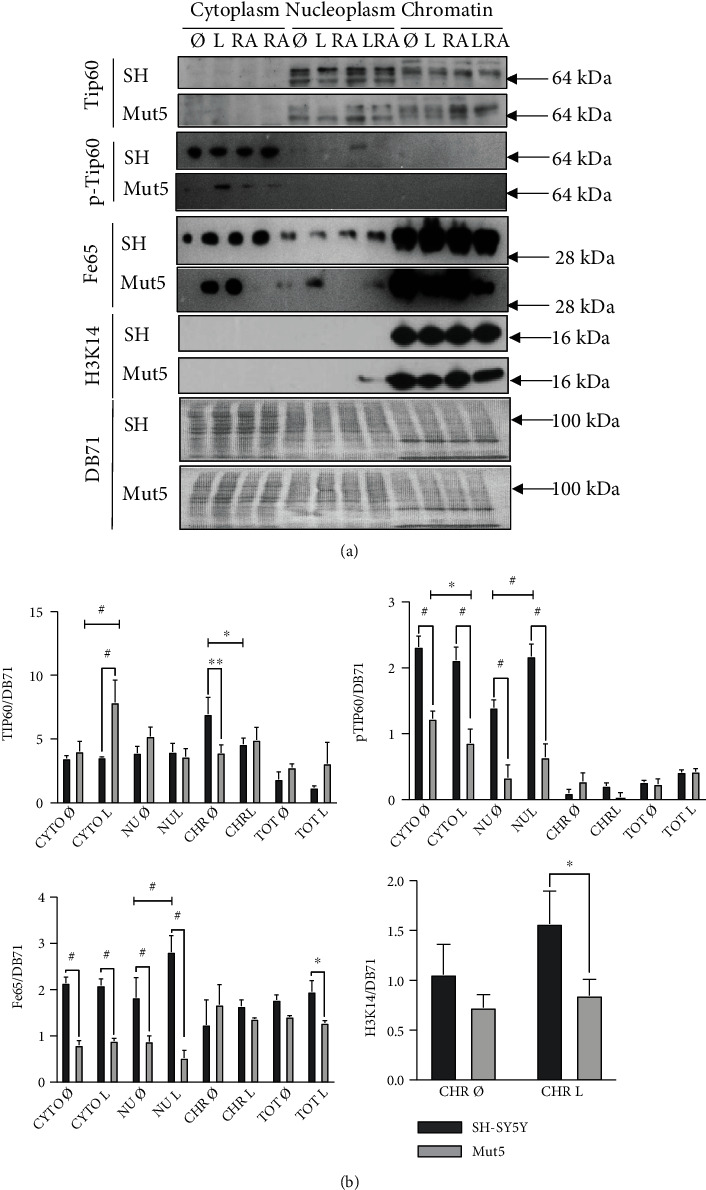
In (a), the SH-SY5Y cells (SH) and Mut5 clone were treated with leptomycin B (L), all-trans retinoid acid (RA), leptomycin B, and RA (LRA) for 30 min, or not, collected and fractionated in cytoplasm (CYTO), nucleoplasm (NU), and chromatin (CHR). Proteins Tip60, phospho-Tip60 (pTip60), Fe65 (C-terminus), and acetylated H3K14 were then analyzed by Western blot. Equal loading was verified by DB71 staining. Overall, we observed higher expression levels of all proteins in the SH-SY5Y cells than in the Mut5 clone. (b) This experiment was repeated in triplicate with or without subcellular fractionation (Total, TOT) but with Fe65 (N- instead of C-terminus) and only the leptomycin B treatment. Statistical analyses mainly revealed higher expressions of the proteins studied in the NU and CYTO fractions of the SH-SY5Y cells, compared to the Mut5 cells. ANOVA with Tukey's multiple comparison test: ^∗^*p* < 0.05; ^∗∗^*p* < 0.01; ^#^*p* < 0.001, *n* = 3.

To investigate the involvement of the Tip60/Fe65 pathway, the intracellular localizations of proteins Tip60, phosphorylated Tip60, Fe65, and histone 3 acetylated at lysine 14 or H3K14 were determined by carrying out Western blots on fractionated SH-SY5Y and Mut5 cells, treated or not with 100 nM leptomycin B for 30 min, in combination with RA, or not (Figure 5(a)). The observed subcellular distributions of the proteins were similar to that of other publications, e.g. [33]. The comparison of the SH-SY5Y and Mut5 cells revealed that these proteins appear to be generally more expressed in the various compartments of the SH-SY5Y than the Mut5 cells. This suggests that this pathway is more active in the SH-SY5Y cells in order to compensate for the higher DSB levels.

This experiment was repeated in triplicate with the leptomycin B treatment only, to obtain quantitative data (Figure 5(b)). Tip60 expression was significantly higher in the chromatin fraction of the untreated SH-SY5Y cells compared to the untreated Mut5 cells. Pospho-Tip60 and Fe65 expressions were significantly more elevated in the cytoplasm and in the nucleoplasm of SH-SY5Y cells compared the Mut5 clone. The acetylated H3K14 protein was higher in the chromatin of the SH-SY5Y compared to the Mut5 cells, but significance was only reached when the cells were treated with leptomycin B in accordance with Figure 5(a).

Overall, the Tip60/Fe65 pathway appears to be more active in the SH-SY5Y than in the Mut5 cells to compensate for longer comet tail lengths, with the leptomycin B increasing the difference of protein expression. However, the fact that pTip60 and Fe65 are statistically not more active in the chromatin fraction of the untreated SH-SY5Y cells goes against this hypothesis. The lower expression levels of these proteins in the Mut5 clone does not explain the lower DSB levels in the Mut clones, compared to the SH-SY5Y cells.

### 3.7. BRCA1, BARD1, and LMO4 Protein Expressions in Subcellular Compartments of the SH-SY5Y and PS1 P117L Cells

**Figure 6:**
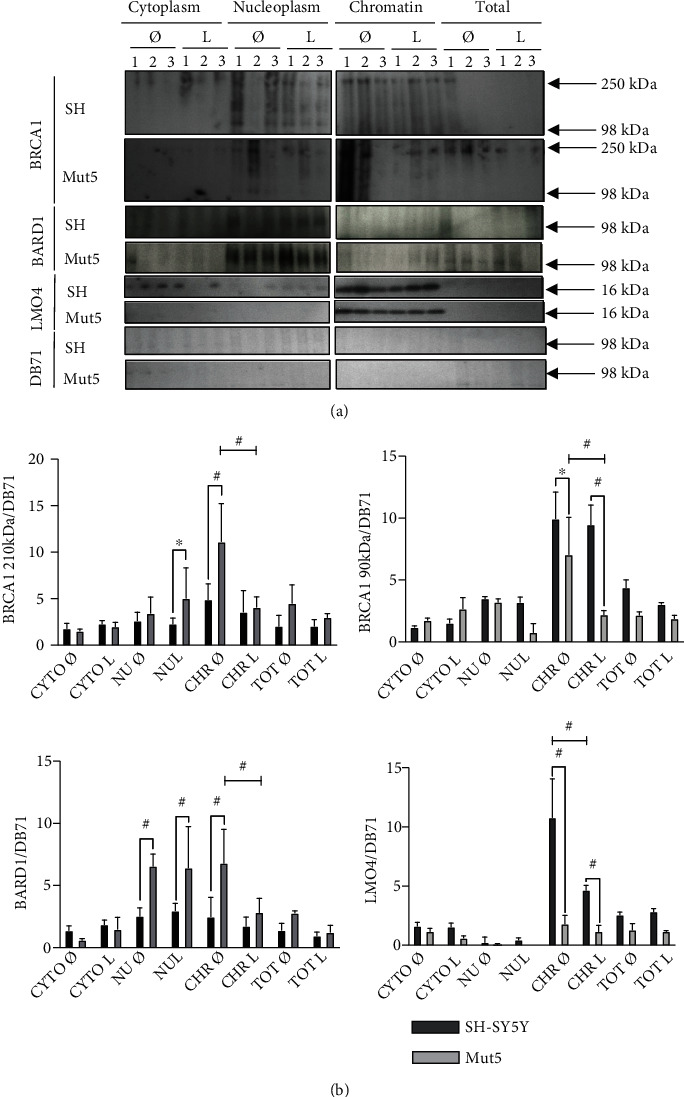
In (a), the SH-SY5Y cells (SH) and Mut5 clone were treated in triplicate (1, 2, and 3) with leptomycin B (L) for 30 min, or not, collected and fractionated in cytoplasm (CYTO), nucleoplasm (NU), and chromatin (CHR), or not (Total, TOT). Proteins BRCA1, BARD1, and LMO4 were then analyzed by Western blot. Equal loading was verified by DB71 staining. In (b), the statistical analyses of the experiment are presented (ANOVA with Tukey's multiple comparison test: ^∗^*p* < 0.05; ^∗∗^*p* < 0.01; ^#^*p* < 0.001, *n* = 3 except for BRCA1 210 kDa and BARD1: *n* = 6). We observed mainly higher expression levels of full-length BRCA1 (210 kDa) and of full-length BARD1 in the chromatin fraction of the Mut5 compared to the SH-SY5Y cells. On the contrary, the 90 kDa BRCA1 isoform and similarly a 110 kDa and a 140 kDa (difference only with leptomycin B) band (statistics not shown), as well as the LMO4 protein, were significantly decreased in expression in the chromatin fraction.

The BRCA1/BARD1 pathway was also investigated to find an explanation for the lower levels of DSBs observed in the Mut clones. Western blots were carried out in triplicate, with fractionated cell lysates to measure the expressions of the BRCA1, BARD1, and LMO4 proteins (Figure 6(a)). We observed a significant increased expression of FL BRCA1 in the chromatin fraction of the Mut5 compared to the SH-SY5Y cells in the absence of leptomycin B and in the nucleoplasm when treated with leptomycin B (Figure 6(b)). On the contrary, the 90 kDa BRCA1 isoform showed a significant decrease of expression in the chromatin fraction of the Mut5 compared to the SH-SY5Y cells, treated or not with leptomycin B. A similar result was observed for the 110 kDa and the 140 kDa (difference only with leptomycin B) bands that were also detected in this size range (data not shown). The FL BRCA1/90 kDa BRCA1 ratio was however significantly increased for the chromatin fraction, independent of the leptomycin B treatment (data not shown). The expression levels of the FL BARD1 protein were like that of the FL BRCA1 protein with significantly increased expressions in the Mut5 compared to the SH-SY5Y cells, in the treated and untreated nucleoplasm and the untreated chromatin. The fact that FL BRCA1 and FL BARD1 proteins are preferentially expressed in the chromatin fraction of the leptomycin B-untreated Mut5, compared to the SH-SY5Y cells, provides a possible explanation for the lower DSB levels in the Mut clones, compared to the wild-type cells. Furthermore, the significant decrease of LMO4 expression in the chromatin compartment of the Mut5, compared to the SH-SY5Y cells, might imply a decreased competition of LMO4 with BARD1 for heterodimerization with FL BRCA1. This would explain the observation of an increased activity of this pathway in the Mut5 clone.

### 3.8. BRCA1 and BARD1 Expressions in the Cortex of B6D2 and PS1 P117L Mice, in the Function of All-Trans Retinoic Acid

**Figure 7:**
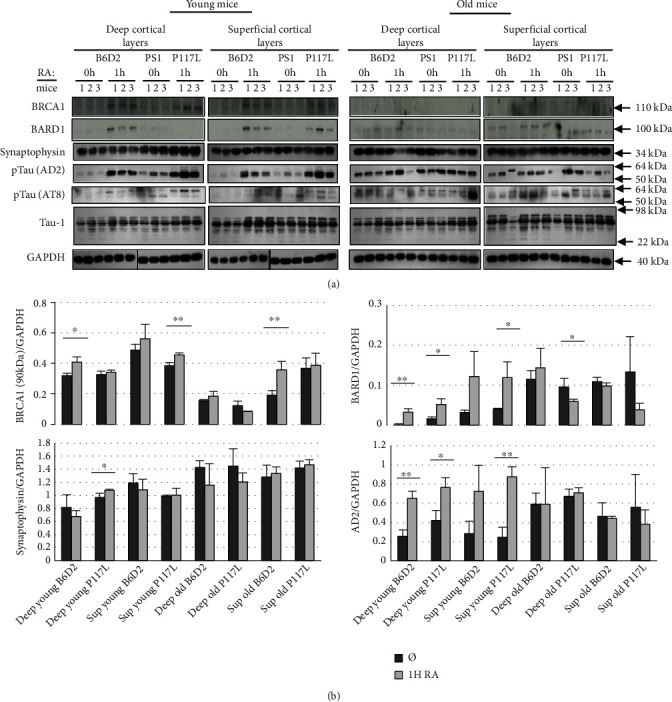
Detection by Western blot (a) of BRCA1 (90 kDa isoform) and full-length (FL) BARD1, synaptophysin, phosphorylated Tau (pTau), and unphosphorylated Tau (Tau-1) in the deep and the superficial (Sup) cortical layers of B6D2 and PS1 P117L young and old male mice, treated or not with all-trans retinoic acid (RA). The full-length BRCA1 protein was below detection level. Three mice (1, 2, and 3) were used each time. The B6D2 and PS1 P117L mice were 1.5- and 17-months-old. Synaptophysin was used as a synaptic marker, pTau (AD2 and AT8) and Tau-1 as cytoskeletal markers, and GAPDH as a loading control. In (b), the protein expressions of BRCA1, BARD1, synaptophysin, and pTau (AD2), visualized in (a), were measured (normalization with GAPDH). Statistically significant differences due to the RA treatment are indicated, ^∗^*p* < 0.05, ^∗∗^*p* < 0.01, *n* = 3.

The neocortical expressions of BRCA1 and BARD1 proteins (Figure 7) were analyzed in B6D2 and PS1 P117L mice of both 1.5 and 17 months (*n* = 3 for each category of age and strain). The hippocampal expressions were also measured and were like the ones of the neocortex. However, these data are not presented due to the lower homogeneity of this tissue compared to cortical layers. We detected the 90 kDa BRCA1 isoform but not the FL isoform. Its expression increased in both the B6D2 and PS1 P117L young mice following a 1 h RA treatment. Signals were weaker in the aged mice (Figure 7(a)). Significant differences were reached, following the RA treatment, in a subset of young mice, i.e., in the deep cortical layers of the B6D2 mice and the superficial cortical layers of the PS1 P117L mice (Figure 7(b)). In both untreated strains, a decreased expression of the 90 kDa BRCA1 isoform was observed with age, except for the PS1 P117L mice in the superior cortical layers.

FL BARD1 expression was increased with RA treatment in all young PS1 P117L and B6D2 mice. In this last case, statistical significance was not reached in the superficial cortical layers. In the aged B6D2 and PS1 P117L mice, increased expression, due to RA, was never observed. However, FL BARD1 expression was significantly higher in the untreated old mice compared to the untreated young ones, except for the PS1 P117L mice in the superior cortical layers. This indicates that aging might influence BARD1 expression.

The expression of synaptophysin remained generally unchanged in the presence or absence of the RA treatment. Nonetheless, there was a significant increased expression with age in the untreated mice, except in the superficial cortical layers of B6D2 mice. Regarding the phospho-Tau protein detected by AD2, its expression increased significantly with RA in both strains, but only in the young mice, except in the superficial cortical layers of B6D2 mice where the increase did not reach significance. Moreover, similarly to the BARD1 expression, AD2 expression increased in all untreated old mice compared to young ones, but not significantly. However, AD2 expression was significantly increased in the deep cortical layers of the B6D2 strain.

Furthermore, the AT8 signals near 50 kDa systematically increased (*p* = 0.004) with age independently of the strain, the cortical layers, and the treatment. This was not the case for the AT8 signal of about 64 kDa or for the Tau-1 signal of about 50 kDa (statistical results not shown). Moreover, there was no significant increase with the RA treatment in all cases.

Overall, these results suggest that both strains do not differ with aging. The RA treatment mainly increased BRCA1 90 kDa and BARD1 FL in the young B6D2 and PS1 P117L mice and both in the deep and superficial cortical layers.

### 3.9. BRCA1-BARD1, BRCA1-LMO4, Fe65-Tip60, and Fe65-pTip60 Heterodimerizations in Subcellular Compartments of the SH-SY5Y and PS1 P117L Cells

**Figure 8:**
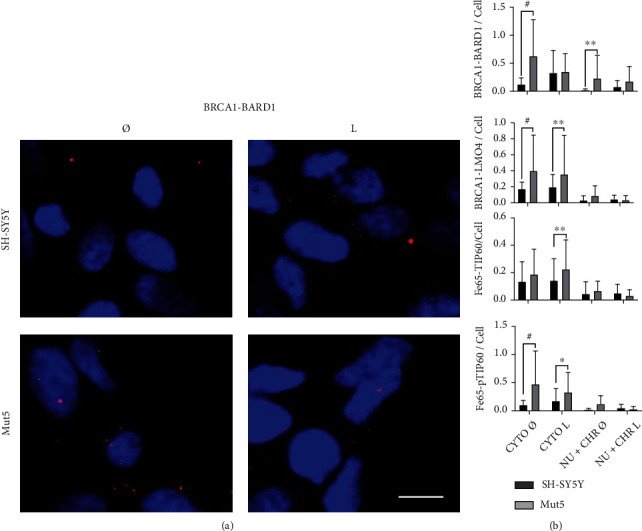
*In situ* proximity ligation assay with the SH-SY5Y and Mut5 cells treated, or not, with leptomycin B (L). In (a), representative pictures of BRCA1-BARD1 heterodimers (red dots) showing higher numbers in the cytoplasm and the nuclei (DAPI staining) of the Mut5 cells compared to the wild-type cells. Scale bar: 75 *μ*M. In (b), graphical representation of the statistical analyses of the experiment for BRCA1-BARD1 heterodimers (*n* = 9). The data analyses for the other heterodimers, BRCA1-LMO4, Fe65-Tip60, and Fe65-pTip60, are also shown (*n* = 9). We observed statistically significant differences (ANOVA with Sidak's multiple comparison test: ^∗^*p* < 0.05; ^∗∗^*p* ≤ 0.002; ^#^*p* ≤ 0.0002) between both strains in the cytoplasm (CYTO), not in the cell nuclei. The nuclei are labelled NU+CHR according to the denomination used for the Western blots (NU: nucleoplasm; CHR: chromatin).

To corroborate the Western blot data, in situ proximity ligation assays were carried out (Figure 8). We observed significantly higher numbers of BRCA1-BARD1 heterodimers in the cytoplasm and in the cell nucleus of the Mut5 compared to the SH-SY5Y cells (Figures 8(a) and 8(b)). This corresponds to the increase of FL BRCA1 and FL BARD1 proteins detected by Western blots. The other heterodimers studied, i.e., BRCA1-LMO4, Fe65-Tip60, and Fe65-pTip60, were increased in the cytoplasm of the Mut5 cells, not in the cell nuclei (Figure 8(b)). Thus, these heterodimers cannot be related to the lower level of DSBs in mutated cells. Furthermore, the statistically significant decreases in the numbers of BRCA1-BARD1 and Fe65-pTip60 heterodimers in the cytoplasm of the Mut5 cells, not in the SH-SY5Y cells, due to the leptomycin B treatment, were not compensated by increases of heterodimers in the nucleus (results not shown). This is consistent with a preferential increase of DSBs in the leptomycin B-treated mutated cells.

### 3.10. ADAM10/*α*-Secretase and BRCA1 siRNA Inhibitions of the SH-SY5Y and PS1 P117L Cells

**Figure 9:**
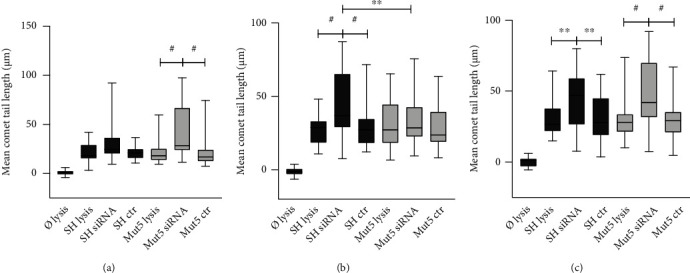
Comet tail lengths (in *μ*M) or DNA double-strand breaks (DSBs) in SH-SY5Y cells (SH) and Mut5 clone after siRNA inhibition, or not (lysis), of ADAM10/*α*-secretase (a, 120 pmol siRNA) and of BRCA1 (b, 120 pmol and c, 240 pmol siRNA). Scrambled sequences were used as negative controls (ctr). We observed a statistically significant increase in DSBs in the Mut5 clone and not in the SH cells with the ADAM10 siRNA and in the SH cells more than in the Mut5 clone with the BRCA1 siRNA. Number of cells measured: 30 < *n* < 32. One-way ANOVA with Bonferroni multiple comparisons test: ^∗^*p* < 0.05; ^∗∗^*p* < 0.002; ^#^*p* < 0.0002.

To demonstrate the relationship between the amyloidogenic pathway and increased DSB production in the PS1 P117L cells compared to the SH-SY5Y cells, we activated this pathway with an siRNA against ADAM10 or *α*-secretase, involved in the antiamyloidogenic pathway (Figure 9(a)). This experiment resulted in a significant increase in DSBs in the Mut5 clone but not in the Wt cells, demonstrating that the amyloidogenic pathway is particularly active in the mutated strain.

In addition, BRCA1 siRNA was used to verify the importance of this repair pathway in the Mut5 clone compared to the Wt cells (Figure 9(b)). A less efficient inhibition was observed in the Mut5 cells than in the Wt cells, consistent with the Western blots data which shows increased BRCA1 expression in the Mut5 cells. However, enhanced inhibition of BRCA1 resulted in a statistically significant increase in DSBs in this strain as well (Figure 9(c)).

## 4. Discussion

### 4.1. Increased Vulnerability towards A*β*_42_ and Decreased Response towards RA due to the Activation of the Amyloidogenic Pathway in the PS1 P117L Cells

It is known that mutations in the *presenilin-1* gene sensitize neurons to DNA damage-induced cell death [43]. DNA damage due to oxidative stress can also induce heterochromatin loss, expression of silent genes, cell cycle reentry, and apoptosis [44]. According to these data, A*β*_42_, by producing DSBs, leads to chromatin relaxation and aberrant gene expression at the origin of dedifferentiation and, if longer treatment times are used, to irreversible damages such as apoptotic cell death.

Our data are consistent with these publications. Indeed, the stimulation of the amyloidogenic pathway, by inhibiting the *α*-secretase with an inhibitor or with an ADAM10 siRNA, produced larger comet tail lengths in the Mut compared to the Wt clones, corroborating their preferential vulnerability towards A*β*_42_. On the contrary, the inactivation of the amyloidogenic cascade, by inhibition of the *β*-, *γ*-, or *β*- + *γ*-secretases, resulted in a consistent decrease of DSBs in the SH-SY5Y and the Wt cells, with shorter comet tail lengths only occasionally observed in the Mut clones. Overall, these data are compatible with those by Chen et al. who observed that the inhibition of the *γ*-secretase increased the *α*-secretase processing of APP and decreased the *β*-secretase processing [45].

Furthermore, the Mut clones were less responsive towards the RA treatment compared to the Wt clones. The simplest explanation for a decreased response towards RA is that of an enhanced activity of the amyloidogenic pathway in the mutated cells. RA, which was shown to decrease the *β*-amyloid cascade via, for instance, enhanced ADAM10/*α*-secretase expression [11], appears to be less effective as a neuroprotective agent in the presence of the *PS1 P117L* mutation.

### 4.2. The Tip60, Fe65, and H3K14 Proteins Are More Expressed in the SH-SY5Y than in the Mutated Cells

We observed lower DSB levels in the untreated Mut clones compared to the untreated wild-type cells as well as in the young PS1 P117L mice compared to young B6D2 mice and less so with aging. As the activation of the amyloidogenic cascade in the Mut clones should result in increased DSBs, we investigated whether the *PS1 P117L* mutation might favor, in parallel to the increase of A*β*_42_, the translocation of Tip60/Fe65 proteins in the nucleus to repair the DSBs. The Fe65 protein would interact with the AICD peptide of 57 amino acids, which is present in the mutated cells, not in the wild-type cells [46]. This explanation was possible as we observed that the leptomycin B treatment, known to inhibit the Tip60/Fe65 pathway, increased DSBs in the Mut clones, more easily than in the Wt clones, similarly to the observations made with the *α*-secretase inhibition. This was also the case for the young PS1 P117L mice. They were more prone to DSB increases due to the leptomycin B treatment than the young B6D2 mice.

We verified, by Western blots, whether the expressions of Tip60, phospho-Tip60, Fe65, and acetylated H3K14 were increased in the nucleus of fractionated Mut compared to control cells. However, we observed a statistically significant decreased expression of the Tip60, the phospho-Tip60, and the Fe65 proteins in the nucleoplasm or chromatin compartments of the Mut5 compared to the SH-SY5Y cells. The leptomycin B was in most cases strengthening the differences observed in the nucleoplasm. This indicates that, contrarily to the investigated hypothesis, the Tip60/Fe65 pathway cannot be responsible for the lower DSB levels in the Mut cells. If increased expressions of pTip60 and Fe65 were observed in the nucleoplasm, this was not the case in the chromatin fraction, making an activation of this pathway in the SH-SY5Y cells unlikely. Furthermore, the in situ proximity ligation assays resulted in no increase of Fe65-Tip60 and Fe65-pTip60 in the nuclei of the mutated cells compared to the SH-SY5Y cells. There was rather an increase of these heterodimers in the cytoplasm of the mutated cells that must however be related to another function than DSB repair. Thus, the Tip60/Fe65 expression levels in the SH-SY5Y and mutated cells, treated with leptomycin B or not, do not explain the higher DSB levels in the wild-type cells, nor the lower DSB levels in the mutated cells.

### 4.3. Lower DSB Levels in the PS1 P117L Cells and Mice Are Related to the Increase of FL BRCA1 and BARD1 in the Chromatin Fraction

The BRCA1 and BARD1 protein expressions were also investigated to provide an explanation to the lower DSB levels in the Mut compared to the wild-type cells. The increased expression of FL BRCA1 and FL BARD1 in the chromatin fraction of the untreated Mut5 clone compared to the SH-SY5Y cells is consistent with the lower DSB levels in the former cells. The in situ proximity ligation assay showed an increase of FL BRCA1-BARD1 heterodimers in the nuclear and cytoplasmic compartments of the Mut5 cells compared to the SH-SY5Y cells. The increased number of heterodimers in the nucleus of the mutated cells is also expected to lower DSB levels. No cytoplasmic retention of the FL BRCA1 was observed in the mutated PS1 P117L cells as was the case for induced pluripotent stem cell-derived familial AD fibroblasts or neurons carrying other PS1 mutations [25, 26]. The lower level of FL BRCA1-BARD1 heterodimers in the SH-SY5Y cells compared to the Mut5 cells, possibly related to lower gene expressions, explains the preferential inhibition of DSBs production with BRCA1 siRNAs in the wild-type cells compared to the Mut5 cells. To deepen our understanding of the implication of this pathway, a next step would be to determine the changes in expression of the different BRCA1 and BARD1 isoforms by quantitative RT-PCR or to target specific alternatively spliced RNAs by siRNAs.

The FL BRCA1 and FL BARD1 protein expressions in the chromatin fraction do not differ significantly between both cell types in presence of leptomycin B. The 90 kDa BRCA1 isoform decreases slightly more in the Mut5 clone than in the SH-SY5Y cells, when treated with leptomycin B. These observations may be due to the facilitated increase of DSBs in Mut, compared to wild-type cells, knowing that the amyloidogenic pathway is more active in the mutated cells.

RA, contrarily to leptomycin B, can diminish DSBs in the wild-type clones preferentially to the mutated clones. RA increases the expressions of the 90 kDa BRCA1 and FL BARD1 proteins in the B6D2 and the PS1 P117L mice. These proteins are involved in DSB repair. These observations align with data showing that *γ*-secretase inhibition increases BRCA1 expression and decreases DSBs, in AD patients [24]. They are also in line with the observations we made that demonstrated in C57BL/6J mice that RA increases the *α*-secretase (ADAM10) and decreases PS1 (*γ*-secretase) and BACE1 (*β*-secretase) [11].

LMO4 expression was decreased in the chromatin fraction of the Mut5 clone, treated or not with leptomycin B. This is concordant with a previous study [47] that showed a decrease of LMO4 expression in hippocampal areas, i.e., the entorhinal cortex (EC) and the CA1 but not the CA3/CA4, of AD compared to control patients. The LMO4 signals were decreased in the cytoplasm as well as in the cell nuclei in AD patients. The RNA for LMO4 was also decreased in the hippocampus of 6-month-old APPsw mice [48]. Moreover, we suggested that the decreased LMO4 expression might represent a model of compensatory neuritogenesis as shown in the SH-SY5Y cells with inhibited LMO4 expression [49, 50]. LMO4 is also known as a BRCA1-interacting protein [31] and might be able to compete with BARD1 for heterodimerization with BRCA1. However, its lower expression in the Mut5, compared to the wild-type cells, rather suggests a diminished competition preventing a decreased DSB repair. In the in situ proximity ligation assays, we observed no statistically significant differences in BRCA1-LMO4 heterodimers in the nuclei of SH-SY5Y compared to mutated cells, and moreover, their numbers were much lower than that of the BRCA1-BARD1 heterodimers, speaking against a model of competition between BARD1 and LMO4, for BRCA1 interaction. However, its impairment of DSB repair might occur differently. The BRCA1-LMO4 heterodimers, by increasing in numbers in the cytoplasm of Mut5 cells compared to the SH-SY5Y cells, as observed by in situ proximity ligation assays, might prevent the increase of BRCA1 in the nuclei and repair of DNA. This might only partly be the case as the BRCA1-LMO4 heterodimers are less numerous than the BRCA1-BARD1 heterodimers and as they do not prevent the increase of these heterodimers in the nucleus.

Overall, we observed that the BRCA1/BARD1 pathway is more active in the untreated mutated cells, compared to the untreated wild-type cells, and can be activated by RA and impaired by leptomycin B.

## 5. Conclusion

Overall, our data shows that the *PS1 P117L* mutation is responsible for a decreased efficiency of RA-dependent DSB repair. Consequently, this observation suggests that RA-mediated neuroprotection strategies might be less effective in familial AD than in nonpathological aging. The RA treatment was shown to increase BRCA1 and BARD1 protein expressions. It is possible that the expression of such RA-dependent genes might be selectively maintained in aging, contributing to a targeted prevention of DSBs by Adaptive Gene Expression (DSB-AGE hypothesis) [11].

An AD model states that at an advanced stage of the disease, BRCA1 is mislocalized to the cytoplasmic NFTs, thus increasing DSBs. In addition, at an earlier stage, there is room for activation of the BRCA1 expression as a compensatory mechanism to decrease DSB levels [24], similarly to what is observed with the PS1 P117L cells or mice which have no or few NFTs. Our study, as previous ones [51, 52], is in favor of such a two-step model of AD progression with a compensatory stage first.

The DSB levels are functionally relevant not only because they are linked to gene expression but also because they may be markers of cellular vulnerability in aging and AD. Moreover, DSB levels depend on ADAM10 or BRCA1 and BARD1. These RA-dependent pathways can be studied as targets for therapeutical strategies.

Principally, our data show that the lower DSB levels in the AD PS1 P117L-mutated cells, compared to the SH-SY5Y cells, cannot be explained by the Tip60/Fe65 pathway but rather by the activation of the BRCA1/BARD1 pathway.

## Data Availability

Data are available on request: armand.savioz@hcuge.ch.
